# Exercise-Induced Cardiac Fatigue in Soldiers Assessed by Echocardiography

**DOI:** 10.3389/fcvm.2021.785869

**Published:** 2021-12-20

**Authors:** Marion Charton, Gäelle Kervio, David Matelot, Thibault Lachard, Elena Galli, Erwan Donal, François Carré, Solène Le Douairon Lahaye, Frédéric Schnell

**Affiliations:** ^1^Department of Cardiology, Pontchaillou Hospital, Rennes, France; ^2^CIC INSERM 1414, CIC-IT, Rennes, France; ^3^LTSI, INSERM, U1099, University of Rennes 1, Rennes, France; ^4^Department of Sport Medicine, Pontchaillou Hospital, Rennes, France; ^5^M2S Laboratory, University of Rennes 2, Rennes, France

**Keywords:** cardiac fatigue, exercise, soldiers, speckle tracking echocardiography, myocardial work

## Abstract

**Background:** Echocardiographic signs of exercise-induced cardiac fatigue (EICF) have been described after strenuous endurance exercise. Nevertheless, few data are available on the effects of repeated strenuous exercise, especially when associated with other constraints as sleep deprivation or mental stress which occur during military selection boot camps. Furthermore, we aimed to study the influence of experience and training level on potential EICF signs.

**Methods:** Two groups of trained soldiers were included, elite soldiers from the French Navy Special Forces (elite; *n* = 20) and non-elite officer cadets from a French military academy (non-elite; *n* = 38). All underwent echocardiography before and immediately after exposure to several days of uninterrupted intense exercise during their selection boot camps. Changes in myocardial morphology and function of the 4 cardiac chambers were assessed.

**Results:** Exercise-induced decrease in right and left atrial and ventricular functions were demonstrated with 2D-strain parameters in both groups. Indeed, both atrial reservoir strain, RV and LV longitudinal strain and LV global constructive work were altered. Increase in LV mechanical dispersion assessed by 2D-strain and alteration of conventional parameters of diastolic function (increase in E/e' and decrease in e') were solely observed in the non-elite group. Conventional parameters of LV and RV systolic function (LVEF, RVFAC, TAPSE, s mitral, and tricuspid waves) were not modified.

**Conclusions:** Alterations of myocardial functions are observed in soldiers after uninterrupted prolonged intense exercise performed during selection boot camps. These alterations occur both in elite and non-elite soldiers. 2D-strain is more sensitive to detect EICF than conventional echocardiographic parameters.

## Introduction

Despite the evident cardio-protective effect of moderate regular aerobic exercise, several studies have questioned the possible deleterious cardiac consequences induced by strenuous long-duration exercise (1–3). Indeed, a transient alteration of ventricular systolic or diastolic function following such exercise has frequently been reported, which is often called exercise-induced cardiac fatigue (EICF) (4–6).

EICF is commonly evaluated with cardiac biomarkers or imaging methods, especially echocardiography. Two-dimensional speckle-tracking echocardiography offers quantitative assessment of myocardial mechanical function and is a more sensitive measure of contractile function than conventional echocardiographic parameters. Transient reductions in left and right ventricular strain (7, 8), and left atrial strain (9) have already been documented after acute endurance exercise. Left ventricular myocardial work (LV MW), analyzed by pressure-strain loops, allows the estimation of myocardial performance. Previous publications demonstrate a relationship between LV MW and LV contractility (10–12) and remodeling (13). Data on this topic are scarce in athletes, and to our knowledge no specific study has addressed the evaluation of LV MW by pressure-strain loops analysis among this population after a prolonged endurance exercise.

Furthermore, most of the studies focused on the acute cardiac effects of a single strenuous long-duration endurance exercise, and less data are available on consequences of repeated bouts of strenuous exercise over several days (5), especially when associated with other constraints as sleep deprivation or mental stress which are an integral part of military selection boot camps.

Lastly, to our knowledge, as most of the studies included well-trained endurance athletes no study has evaluated the potential occurrence of EICF in population presenting with different experience and training.

The aim of the present study was to identify and to quantify the cardiac effects of a several days of uninterrupted strenuous exercise during military selection boot camps, and to study the influence of prior experience and training level.

## Materials and Methods

### Study Population

Two populations of French soldiers were prospectively included:

- A group of non-elite soldiers: second-year officer cadets from a French military academy (*n* = 39), the Special Military School of Saint-Cyr, located in Coëtquidan, Brittany. This was their first selection boot camp, their previous selection was solely based on their academic skills. Their regular physical training included 3 weekly 2 h sessions combining endurance and resistance activities.
- A group of elite soldiers: French Navy Special Forces soldiers (*n* = 20), located in Lorient, Brittany. This group was more experienced, as they had already been selected during previous boot camps to be able to join this elite unit. This unit is considered one of the world's references in the field of Special Forces and counterterrorism units. Therefore, their selection is extremely tough, and they all have a very high level of physical training and a great individual's ability to adapt to a hostile environment.

All participants gave written informed consent to participate in this study, which received the approval of the local Rennes University Hospital and regional Ethics Committee (Number 35RC13_8801) and was conducted in accordance with the “Good Clinical Practice” Guidelines as laid down in the Declaration of Helsinki.

### Experimental Procedure

#### Boot Camp

All subjects participated in a winter boot camp, which consisted of intense and continuous outdoor endurance and resistance physical practice. The boot camps were part of the usual military preparation, we were therefore not able to change them. The soldiers had to undergo several timed orienteering marches, abseiling, crossing, and swimming exercises. This was associated with difficult outdoor living conditions, sleep deprivation (only a few hours of sleep during the boot camp), and mental stress. In both groups the goal of the boot camp was to push the soldiers to their very limit, in order to perform a selection of the soldiers. As the experience and training level of both groups of soldiers were completely different, the level of difficulty and duration of the boot camp were also different (respectively, 36 and 96 h). The programs were specific to each Army corps and adapted to the performance level of the subjects by experienced military instructors, which kept the soldier constantly under pressure.

All subjects were healthy, this was confirmed prior to inclusion by a pre-participation health screening, including clinical examination and resting ECG. Height, weight, resting heart rate, and blood pressure were recorded. They all were non-smokers, with no cardiovascular risk factor, no history of cardiopulmonary, no metabolic, nor neuromuscular disorders, and were not taking any chronic medication.

#### Echocardiography

Echocardiographies were recorded the day before and immediately following the end of the boot camp, using a Vivid Q (GE Healthcare, Horten, Norway). Offline analysis was performed on a dedicated workstation (EchoPAC Version 202, GE Vingmed Ultrasound, Norway).

**Figure 1:**
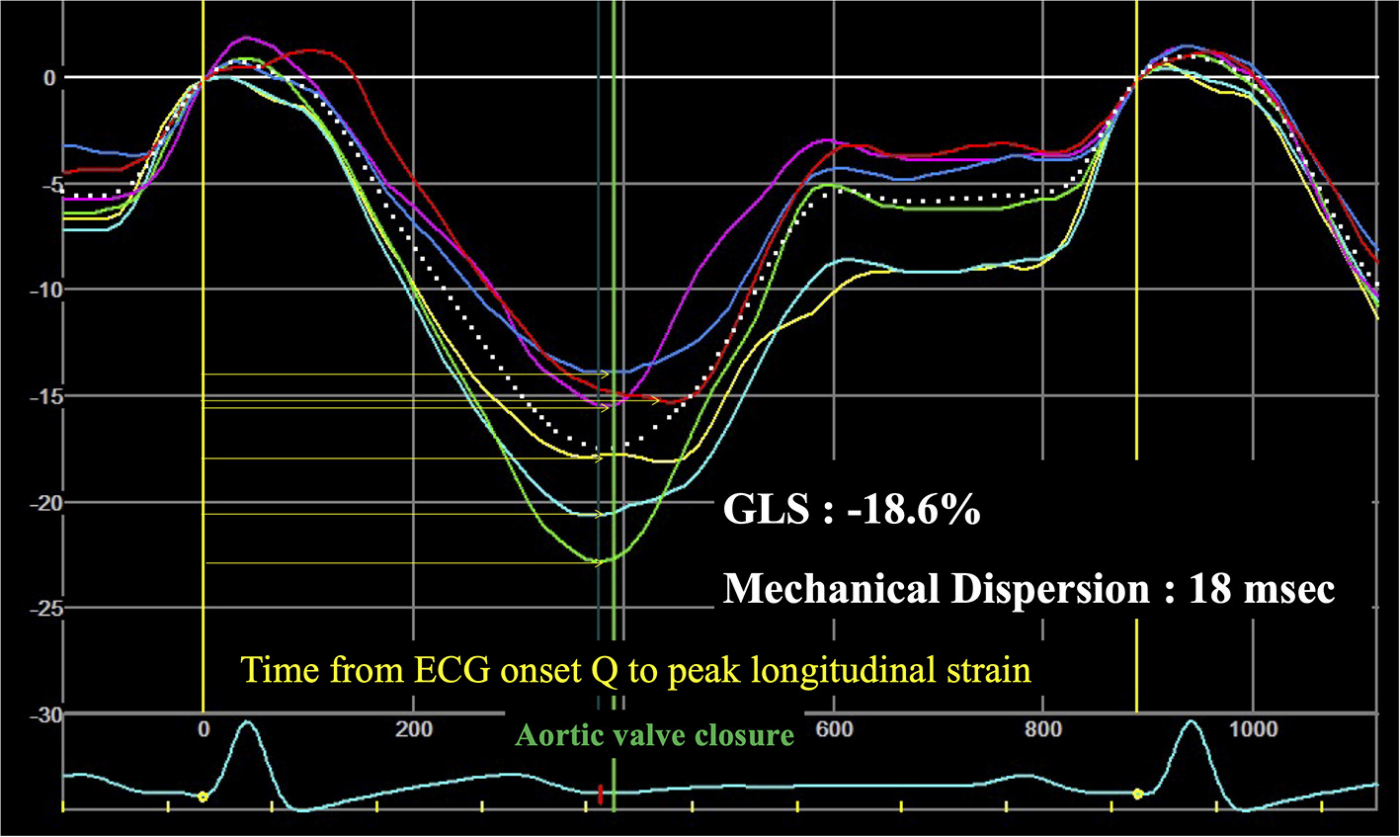
Example of speckle-tracking strain analysis of left ventricular mechanical dispersion. GLS, global longitudinal strain; Mechanical Dispersion, assessed by the standard deviation of the 18 segments (for the clarity of the figure only 6 segments are shown).

LV cardiac chamber size, LV systolic and diastolic function were assessed as recommended (14, 15). LV global longitudinal strain (GLS) was assessed on the 18 segments obtained from two-dimensional grayscale images acquired in the apical 4, 3, and 2 chamber views at a frame rate ≥60 frames/s (16). LV Mechanical dispersion was calculated as the standard deviation of the time to maximal myocardial shortening, measured from the electrocardiographic onset Q/onset R- wave in the 18 LV segments (Figure 1) (17).

Right ventricular (RV) morphological parameters were assessed as recommended, using RV end diastolic (ED) basal and mid cavity diameter, RVED, and RVES area (13). RV conventional parameters of systolic function were assessed using TAPSE, s' tricuspid wave and RV fractional area change (RV FAC). RV GLS was determined as the average of the 3 RV free wall segments, from an apical four-chamber image focused on the RV (18).

Left atrial (LA) and right atrial (RA) volumes were measured using the modified Simpson's method from the 4 and 2 chamber views for the LA and 4 chamber views for the RA. Atrial longitudinal strain was assessed for left atrium on the 12 segments of the 4- and 2-chamber views, and for right atrium on the six segments of apical four-chamber view as recommended (18).

**Figure 2:**
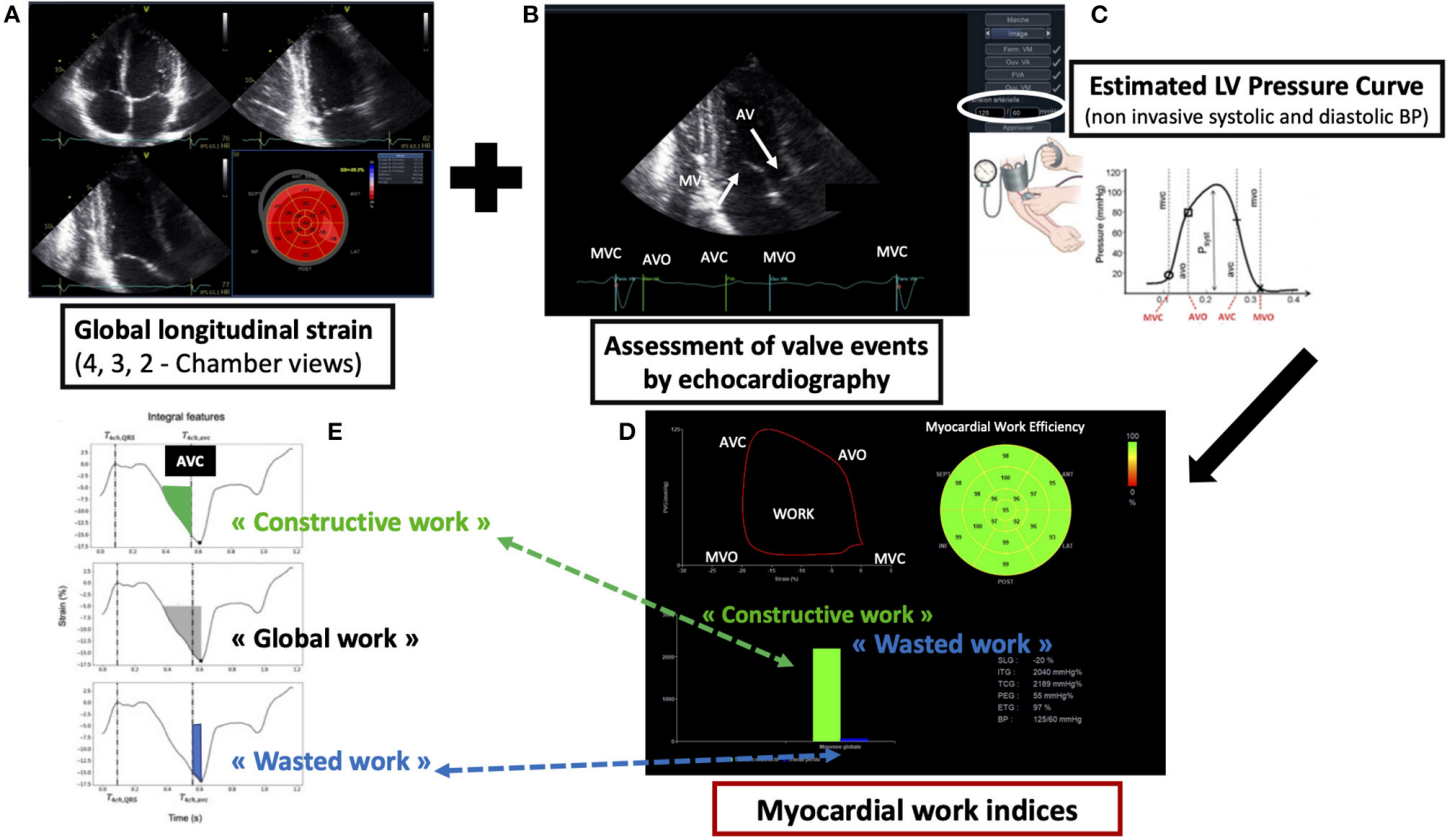
Methodology of myocardial work (MW) assessment. **(A)** Acquisition of apical four-, two-, and three-chamber view and evaluation of global longitudinal strain (GLS) with left ventricular (LV) bull's eye. **(B)** Assessment of valve events by echocardiography (MVC, mitral valve closure; AVO, aortic valve opening; AVC, aortic valve closure; MVO, mitral valve opening). **(C)** Introduction of systolic and diastolic blood pressure; peak systolic LV pressure is assumed equal to the peak arterial pressure recorded from the brachial cuff systolic pressure. A patient-specific LV pressure curve is constructed by the software, adjusting LV pressure curve to the duration of the isovolumic and ejection phases, defined by valvular timing events. Strain and pressure data are synchronized using the R wave on ECG as common time reference. **(D)** Evaluation of global values of all myocardial work (MW) components; bars showing the constructive (green bar) and wasted (blue bar) global work. **(E)** During the isovolumic contraction and LV ejection period, segmental shortening contributes to the final LV ejection, whereas segmental stretch or lengthening does not contribute to LV ejection. As a result, the work performed by the myocardium during segmental shortening represents constructive work (CW), whereas the work performed by the myocardium during stretch or segmental lengthening represents energy loss, defined as wasted work (WW).

LV MW and related indices were estimated by the combination of LV strain data and the non-invasive estimation of the LV pressure curve as previously described (19). Briefly, the peak systolic LV pressure was assumed equal to the peak arterial pressure recorded from the brachial cuff systolic pressure. A patient-specific LV pressure curve was then constructed by the software (EchoPAC, GE Vingmed Ultrasound, Norway), adjusting LV pressure curve to the duration of the isovolumic and ejection phases, defined by valvular timing events. Strain and pressure data were synchronized using the R wave on ECG as common time reference. The area within the peak segmental longitudinal strain provided an index of LV MW for each myocardial segment. During the isovolumic contraction and LV ejection period, segmental shortening contributes to the final LV ejection, whereas segmental stretch or lengthening does not contribute to LV ejection. As a result, the work performed by the myocardium during segmental shortening represents constructive work (CW), whereas the work performed by the myocardium during stretch or segmental lengthening represents energy loss, defined as wasted work (WW). So, the CW was defined as MW during segmental shortening in systole, and segmental lengthening during the isovolumic relaxation time. The WW was defined as the work performed during lengthening in systole and shortening in isovolumic relaxation, associated with energy loss. By averaging segmental CW and WW for each segment, LV global constructive work (LV GCW) and LV global wasted work (LV GWW) were estimated for the entire LV (19) (Figure 2).

Both inter-observer and intra-observer concordances for the estimation of CW and WW work have already been evaluated in a previous study from our research group (20).

### Statistical Analyses

Normal distribution of all continuous variables was confirmed by a Kolmogorov-Smirnov test. All data were expressed as means and standard deviations. Quantitative data were compared using Student's *t*-test. Paired student's *t*-tests were used to determine differences between pre- and post-exercise values for the various echocardiographic parameters. Student's *t*-tests were used to determine differences between non-elite and elite groups. The response from pre-to-post-exercise in the different groups was compared using repeated measures ANOVA with time of echocardiography (pre- vs. post- exercise) as within-subject effect and group (elite vs. non-elite soldiers) as a between-subject effect. Level of significance was set to *p* < 0.05. Statistical analyses were conducted using the SPSS (v.20 SPSS Inc.; Chicago, IL).

## Results

One subject from the non-elite group was excluded because he got injured and was not able to complete the follow-up echocardiography. Therefore, finally 58 soldiers, 20 in the elite group, and 38 in the non-elite group completed the study.

Subject characteristics are shown in Table 1. The subjects in the elite group were older than those in the non-elite group (respectively, 26.6 ± 3.4 vs. 21.7 ± 0.6 years, *p* < 0.0001). There was no difference in body surface area between both groups, even if subjects in the elite group were heavier (78.0 ± 9.0 vs. 73.1 ± 8.5 kg, *p* = 0.05) with a higher body mass index (24.2 ± 1.5 vs. 22.8 ± 1.9 kg.m^−2^, *p* = 0.006). The subjects in the non-elite group lost weight during the boot camp (73.1 ± 8.5 kg before-exercise vs. 72.4 ± 7.9 kg post-exercise, *p* = 0.001). No difference in systolic blood pressure (SBP) between groups was observed both before and after exercise. The diastolic blood pressure (DBP) was slightly lower in the non-elite group both before (*p* < 0.0001) and after (*p* = 0.005) exercise. Exercise-induced decrease of SBP was only observed in the non-elite group (121.2 ± 10.2 vs. 113.5 ± 10.6 mmHg; *p* = 0.001, respectively, before and after exercise) (*p* = 0.032 for interaction), without any change in DBP. Heart rate demonstrated a significant overall increase following exercise (63.9 ± 10.5 vs. 81.8 ± 11.1 bpm, *p* < 0.0001 in the elite group; 58.8 ± 8.5 vs. 70.3 ± 12.7 bpm, *p* < 0.0001 in the non-elite group, respectively, before and after exercise), with a higher increase (*p* = 0.031 for interaction) and a higher HR after exercise in the elite group.

**Table 1:** Clinical characteristics of the study populations.

	**Elite group**			**Non-elite group**			**Comparison between**		**Interaction**
	**(*****n** **=*** **20)**			**(*****n** **=*** **38)**			**two groups**		
	**Pre-**	**Post-**	**Pre/post-**	**Pre-**	**Post-**	**Pre/Post-**	**Pre-exercise**	**Post-exercise**	***p*-value**
	**exercise**	**exercise**	***p*-value**	**exercise**	**exercise**	***p*-value**	***p*-value**	***p*-value**	
Age (years)	26.6 ± 3.4		–	21.7 ± 0.6		–	<0.0001		
BSA (m^2^)	1.97 ± 0.15	–	–	1.90 ± 0.14	1.89 ± 0.12	0.607	0.109	–	
Weight (kg)	78.0 ± 9.0	–	–	73.1 ± 8.5	72.4 ± 7.9	0.001	0.05	–	
BMI (kg.m^−2)^	24.2 ± 1.5	–	–	22.8 ± 1.9	22.5 ± 1.8	0.001	0.006	–	
HR (bpm)	63.9 ± 10.5	81.8 ± 11.1	<0.0001	58.8 ± 8.5	70.3 ± 12.7	<0.0001	0.052	0.001	0.031
SBP (mmHg)	118.1 ± 8.3	117.9 ± 7.7	0.929	121.2 ± 10.2	113.5 ± 10.6	0.001	0.267	0.111	0.032
DBP (mmHg)	73.4 ± 10.5	71.7 ± 7.3	0.523	62.0 ± 6.9	64.3 ± 9.9	0.218	<0.0001	0.005	0.215

*BSA, body surface area; BMI, body mass index (kg.m^−2^); HR, heart rate (bpm); SBP, systolic blood pressure (mmHg); DBP, diastolic blood pressure (mmHg)*.

Echocardiographic data before and after boot camp are shown in Tables 2–4. Prior to exercise, no abnormality was detected on the resting echocardiography or ECG performed.

**Table 2:** Left ventricular echocardiographic parameters.

	**Elite group**			**Non-elite group**			**Comparison between**		**Interaction**
	**(*****n** **=*** **20)**			**(*****n** **=*** **38)**			**two groups**		
	**Pre-**	**Post-**	**Pre/post-**	**Pre-**	**Post-**	**Pre/Post-**	**Pre-exercise**	**Post-exercise**	***p*-value**
	**exercise**	**exercise**	***p*-value**	**exercise**	**exercise**	***p*-value**	***p*-value**	***p*-value**	
LVED vol. (ml)	117.3 ± 23.6	126.3 ± 21.1	0.031	100.8 ± 17.9	102.9 ± 20.5	0.373	0.004	<0.0001	0.116
LVES vol. (ml)	41.3 ± 9.5	43.6 ± 10.3	0.261	34.3 ± 7.8	34.2 ± 7.5	0.872	0.004	<0.0001	0.210
LVEF (%)	64.8 ± 4	65.7 ± 4.2	0.511	66.0 ± 4.0	66.7 ± 3.6	0.327	0.256	0.309	0.878
Mean S' (cm.s^−1^)	10.7 ± 1.3	11.1 ± 1.5	0.379	10.9 ± 1.1	11.0 ± 1.4	0.654	0.591	0.697	0.549
LV GLS (%)	−21.2 ± 1.5	−20.0 ± 1.7	0.02	−21.6 ± 1.5	−20.2 ± 1.4	<0.0001	0.348	0.652	0.575
Mechanical dispersion (ms)	23.2 ± 8.0	27.2 ± 7.3	0.094	20.9 ± 6.8	29.1 ± 6.1	<0.0001	0.243	0.306	0.093
LV GCW (mmHg%)	2179.2 ± 236.6	1780.6 ± 527.7	0.048	2231.2 ± 302.5	1862.8 ± 279.3	<0.0001	0.219	0.860	0.850
LV GWW (mmHg%)	70.5 ± 21.2	79.1 ± 31.1	0.354	70 ± 27.6	60.2 ± 28.8	0.182	0.551	0.114	0.131
E (m.s^−1^)	0.78 ± 0.16	0.77 ± 0.14	0.758	0.79 ± 0.13	0.81 ± 0.14	0.207	0.906	0.230	0.340
A (m.s^−1^)	0.39 ± 0.09	0.38 ± 0.08	0.803	0.35 ± 0.08	0.43 ± 0.11	<0.0001	0.090	0.129	0.022
DTI (ms)	217.0 ± 35.0	197.4 ± 43.0	0.160	221.6 ± 1.7	193.6 ± 43.1	0.003	0.547	0.766	0.610
E/A	2.05 ± 0.46	2.07 ± 0.42	0.890	2.33 ± 0.58	1.96 ± 0.46	0.004	0.062	0.567	0.072
E/e'	4.45 ± 1.39	4.76 ± 0.61	0.391	4.51 ± 0.94	4.99 ± 0.95	0.01	0.764	0.297	0.568
e' (cm.s^−1^)	16.8 ± 0.21	16.1 ± 0.29	0.309	17.8 ± 0.23	16.5 ± 0.21	0.01	0.135	0.527	0.514

*LV, left ventricle; LVED vol,: LV end-diastolic volume (ml); LVES vol., LV end-systolic volume (ml); LVEF, Left ventricular ejection fraction (%); GLS, global longitudinal strain (%); GCW, global constructive work (mmHg%); GWW, global wasted work (mmHg%); E, Peak E mitral velocity (m.s−1); A, Peak A mitral velocity (m.s−1); DTI, E-wave deceleration time (ms); e', early diastolic velocity (cm.s^−1^)*.

**Table 3:** Right ventricular echocardiographic parameters.

	**Elite group**			**Non-elite group**			**Comparison between**		**Interaction**
	**(*****n** **=*** **20)**			**(*****n** **=*** **38)**			**two groups**		
	**Pre-**	**Post-**	**Pre/post-**	**Pre-**	**Post-**	**Pre/Post-**	**Pre-exercise**	**Post-exercise**	***p*-value**
	**exercise**	**exercise**	***p*-value**	**exercise**	**exercise**	***p*-value**	***p*-value**	***p*-value**	
RVED basal diameter (cm)	3.9 ± 0.5	3.7 ± 0.6	0.337	3.6 ± 0.4	3.7 ± 0.4	0.2	0.050	0.835	0.103
RVED mid cavity diameter (cm)	3.2 ± 0.5	3.1 ± 0.6	0.257	2.8 ± 0.4	2.8 ± 0.3	0.79	0.001	0.068	0.193
RVED area (cm^2^)	16.8 ± 2.3	16.1 ± 3.6	0.301	15.4 ± 2.8	15.0 ± 2.5	0.364	0.059	0.184	0.628
RVES area (cm^2^)	8.5 ± 1.8	8.3 ± 1.8	0.554	7.6 ± 1.2	7.2 ± 1.6	0.072	0.039	0.031	0.641
RVFAC (%)	49.5 ± 7.6	48.3 ± 6.8	0.625	49.6 ± 6.7	51.9 ± 7.5	0.099	0.943	0.081	0.174
TAPSE (mm)	23.7 ± 4.5	24.9 ± 3.7	0.296	24.6 ± 2.8	25.3 ± 2.8	0.228	0.423	0.646	0.691
S'tric (cm.s^−1^)	14.4 ± 2.1	14.8 ± 2.6	0.560	14.8 ± 0.2	14.9 ± 0.2	0.710	0.476	0.770	0.793
Free wall longitudinal strain (%)	−28.0 ± 4.0	−25.3 ± 3.9	0.003	−30.5 ± 2.8	−27.8 ± 3.5	<0.0001	0.006	0.017	0.917
LB (%)	−25.1 ± 5.8	−25.1 ± 9.2	0.978	−26.0 ± 3.9	−24.5 ± 4.9	0.155	0.388	0.782	0.455
LM (%)	−30.2 ± 5.2	−26.7 ± 6.7	0.007	−31.6 ± 3.7	−29.4 ± 4.2	0.006	0.176	0.122	0.367
LA (%)	−28.8 ± 5.6	−24.5 ± 6.4	0.037	−32.9 ± 4.9	−30.2 ± 5.6	0.016	0.007	0.001	0.455

*RV, right ventricle; ED, end diastolic; ES, end systolic; RVFAC, RV fractional area change (%); TAPSE, tricuspid annular plane systolic excursion (mm); S'tric, s tricuspid wave velocity (cm.s^−1^); LB, latero-basal segment; LM, latero-mid segment; LA, latero-apical segment*.

**Table 4:** Atrial parameters.

	**Elite group**			**Non-elite group**			**Comparison between**		**Interaction**
	**(*****n** **=*** **20)**			**(*****n** **=*** **38)**			**two groups**		
	**Pre-**	**Post-**	**Pre/post-**	**Pre-**	**Post-**	**Pre/Post-**	**Pre-exercise**	**Post-exercise**	***p*-value**
	**exercise**	**exercise**	***p*-value**	**exercise**	**exercise**	***p*-value**	***p*-value**	***p*-value**	
LA volume (ml)	40.8 ± 11.3	45.9 ± 13.3	0.103	34.0 ± 11.6	36.7 ± 12.9	0.137	0.072	0.025	0.459
LA Peak atrial longitudinal strain (%)	56.3 ± 4.4	51.9 ± 6.1	0.003	59.6 ± 10.1	51.0 ± 10.1	<0.0001	0.088	0.689	0.091
RA volume (ml)	46.4 ± 15.4	42.1 ± 11.9	0.189	42.8 ± 11.3	45.6 ± 10.4	0.088	0.316	0.246	0.029
RA Peak atrial longitudinal strain (%)	55.5 ± 8.2	49.4 ± 8.8	<0.0001	59.8 ± 9.5	52.0 ± 9.8	<0.0001	0.337	0.374	0.089

*LA, left atrium; RA, right atrium*.

The LVED and LVES volumes were larger in the elite group than in the non-elite group, both before as well as after the boot camp (this was also the case when considering the BSA indexed values; Table 2). An exercise-induced increase in LVED volume was only observed in the elite group, nevertheless this exercise increase was not significantly different between both groups (*p* = 0.116 for interaction). LVEF and mean s' mitral wave were not different between both groups, and these parameters were not altered after exercise. No inter-group difference in LV longitudinal strain, mechanical dispersion, LV GCW or LV GWW was observed before and after exercise. However, after exercise, we noted an intra-group reduction of LV longitudinal strain (−21.2 ± 1.5 vs. −20.0 ± 1.7%, *p* = 0.02 in the elite group and −21.6 ± 1.5 vs. −20.2±1.4%, *p* < 0.0001 in the non-elite group) and of LV GCW (2179.2 ± 236.6 vs. 1780.6 ± 527.7 mmHg%, *p* = 0.048 in the elite group and 2231.2 ± 302.5 vs. 1862.8 ± 279.3 mmHg%, *p* < 0.0001 in the non-elite group) whereas LV GWW was unchanged. After exercise, the mechanical dispersion increased only in the non-elite group (23.2 ± 8.0 vs. 27.2 ± 7.3 ms, *p* = 0.094 in the elite group; 20.9 ± 6.8 ms vs. 29.07 ± 6.09 ms, *p* < 0.0001 in the non-elite group), nevertheless this exercise increase was not significantly different between both groups (*p* = 0.093 for interaction).

As regards to the right ventricle, prior to exercise RVED basal and mid cavity diameter, and RV systolic area were larger in the elite group than in non-elite one (Table 3). No intra-group exercise-induced change in RV dimensions was noted. There was no difference between groups, and no exercise-induced change for conventional right ventricle systolic parameters (RVFAC, TAPSE, and s' tricuspid wave) (Table 3). Whereas, due to a lower strain value in the apical segment the longitudinal RV strain of the free wall was lower in the elite than in the non-elite both at baseline and post-exercise; an exercise-induced reduction was observed in both population (−28.0 ± 4.0 vs. −25.3 ± 3.9%, *p* = 0.003 in the elite group and −0.5 ± 2.8 vs. −27.8 ± 3.5%, *p* < 0.0001 in the non-elite group). These exercise-induced alterations were only present in the mid and apical segments, with a preservation of the basal segment.

No inter-group difference was observed for conventional parameters of LV diastolic function before and after exercise (Table 2). Nevertheless, diastolic function was significantly affected by exercise only in the non-elite group with a decrease in E wave deceleration time (221.6 ± 1.7 vs. 193.6 ± 43.1 ms, *p* = 0.003), E/A ratio (2.33 ± 0.58 vs. 1.96 ± 0.46, *p* = 0.004), and e' value (17.8 ± 0.23 vs. 16.5 ± 0.21 cm.s^−1^, *p* = 0.01) and an increase in peak A velocity (0.35 ± 0.08 vs. 0.43 ± 0.11 m.s^−1^, *p* < 0.0001) and E/e' ratio (4.51 ± 0.94 vs. 4.99 ± 0.95, *p* = 0.01). Only the exercise-induced increase in peak A velocity was significantly different in both groups (*p* = 0.022 for interaction).

With regards to the atria, no inter-group difference in volumes and functions was observed before exercise (Table 4). The volumes of both atria were not significatively affected by exercise, but the exercise-induced changes in RA volume were opposite (*p* = 0.029 for interaction) with a non-significant decrease in RA volume in elite soldiers and a non-significant increase in non-elite soldiers. An exercise-induced decrease in atrial reservoir strain was observed in both groups (LA longitudinal strain: 56.3 ± 4.4 vs. 51.9 ± 6.1%, *p* = 0.003 in the elite group and 59.6 ± 10.1 vs. 51.0 ± 10.1%, *p* < 0.0001 in the non-elite group; RA longitudinal strain: 55.5 ± 8.2 vs. 49.4 ± 8.8%, *p* < 0.0001 in the elite group and 59.8 ± 9.5 vs. 52.0 ± 9.8%, *p* < 0.0001 in the non-elite group).

## Discussion

Our study aimed to assess the effect of a boot camp with several days of uninterrupted strenuous endurance and resistance exercise associated with sleep deprivation and mental stress, in two populations of soldiers with different training level. After the boot camp we observed an alteration in ventricular systolic and diastolic functions in both studied populations. 2D-Strain seems more sensitive to detect EICF than conventional echocardiographic parameters. The alterations occurred in both groups, nevertheless some alterations occurred solely in non-elite group, which raises the question of a potential protective effect of training.

Chronic endurance training induces a physiological cardiac remodeling characterized by a harmonious dilation of the cardiac chambers, and a slight reduction in resting ventricular function (21). This increase in cardiac volumes and mass enables a greater oxygen consumption during exercise (22). Therefore, as expected, the more trained elite group demonstrate a more important cardiac remodeling than the non-elite one. Indeed, the elite group had larger LV ED and ES volumes, RVED basal and mid cavity diameter as compared to the non-elite. Furthermore, as previously described in endurance athletes (23), the peak global longitudinal RV strain was lower in the elite group than in non-elite one (−28.0 ± 4.0 vs. −30.5 ± 2.8%; *p* = 0.006).

### Exercise-Induced Cardiac Fatigue

Our results are in accordance with previous studies which demonstrated frequent alterations in systolic and diastolic functions in response to strenuous acute endurance exercise (6, 7). Even if these alterations did not concern all subjects (24), and was not reported in all studies (25), the post-exercise values reduction seemed to be related to the duration of exercise (2). Therefore, due to the intense and prolonged nature of our exercise protocols, these results were expected.

In both groups we didn't demonstrate any alteration of conventional markers of LV and RV systolic function after boot camp. Indeed, there was no change in LVEF or mean mitral s' wave, TAPSE, RV FAC, and s' tricuspid wave. But we noted a decrease of more subtle markers of LV and RV systolic function as global LV longitudinal strain and global CW analyzed by pressure-strain loops. The global RV free wall longitudinal strain was also decreased, due to an alteration in the mid and apical segments. This discrepancy between the basal segment and the mid/apical segments of the RV free wall, was already demonstrated in a previous study (26), although the mechanisms involved remain to be elucidated.

Both left and right atrial function were also altered with a decrease of their peak longitudinal strain, which might be considered as a subtle alteration of left and right diastolic function (27). The clinical relevance of these changes is still unknown. While it is known, that reduction of atrial strain in master athletes is associated with lone paroxysmal atrial fibrillation (PAF) (28, 29), the fact that this transient reduction in atrial reservoir function is related to an increased risk of occurrence of PAF later remains to be determined. We were unable to provide data on the recovery of this diastolic alteration. However, all elite subjects who were regularly exposed to the same type of endurance exercise had normal baseline echocardiographic parameters. Furthermore, none of the post-exercise values was considered as pathological. Lastly previous studies which have performed follow up echocardiography reported a diastolic function recovery after a period of 28 h (1).

### Impact of Training Level

The comparison between both groups of soldiers is challenging, as they did not undergo the same exercise protocol. Nevertheless, both underwent programs adapted to their abilities and training level; the goal was to push them to their very limit. The boot camp used in the elite group was more intense and more prolonged than the one used in the non-elite group (96 vs. 36 h). Therefore, we might have expected a more important alteration of systolic and diastolic parameters in the elite group. Indeed, in previous studies the greatest reduction in post-exercise values was reported in athletes who completed the longest events (2, 24). Our results showed the opposite, mechanical dispersion and usual parameters of diastolic function were only altered in the non-elite group.

After the boot camp we observed an alteration in both studied populations. Indeed, there was a significant decrease in the strain values of the LV, RV, LA, and RA. Furthermore, the comparison between the exercise-induced changes in the different parameters between elite and non-elite groups were not significantly different. Nevertheless, there were some slight differences in the exercise induced changes that might suggest a more important alteration in the non-elite group.

As regards to systolic function, while 2D-strain and myocardial work parameters decreased after exercise in both groups, we observed that mechanical dispersion of LV strain only increased in the non-elite group. An increase in electro-mechanical delay post-exercise has been previously reported, suggesting some degree of LV mechanical discoordination after intense exercise (30). Mechanical dispersion assessed by two-dimensional strain reflects heterogeneous myocardial contraction. We have previously demonstrated that mechanical dispersion is a good marker to differentiate a remodeling from pathological and physiologic origin (17). Furthermore, mechanical dispersion was related to ventricular arrhythmia in post-myocardial infarction patients (31). Nevertheless, further studies are needed to determine if this exercise induced alteration of mechanical dispersion may represent a long-term arrhythmogenic substrate.

As regards to diastolic function, while LA and RA peak longitudinal strain decreased after exercise in both groups, the usual parameters of diastolic function were only altered after exercise in the non-elite group, with a decrease in E/A (as a result of an increase in A wave), E wave deceleration time and e' and an increase in E/e'. These alterations may represent a more marked stage of diastolic dysfunction, the early one being only represented by the alteration of atrial strain. Exercise-induced diastolic dysfunction might be partially explained by a reduction in preload. Indeed, the same findings were demonstrated previously after a marathon, and preload augmentation through passive leg elevation (PLE) corrected some of these alterations (32). But although e' increased with post-exercise PLE, it did not reach pre-exercise supine levels, and did not correct the increase of A wave post exercise, suggesting that some intrinsic impairment in myocardial relaxation and compliance may persist despite normalization of preload (32). The impact of a potential reduction of preload due to the dehydration associated with exercise might also be raised by our results. But, in disfavor of this hypothesis, we noted no decrease in LVED volume in both groups, but an increase in the elite group and a trend of increase in non-elite. Moreover, all subjects could hydrate without restriction during exercise.

### A Protective Effect of Training?

As regards to the results of our study, we can speculate that appropriate training prior to strenuous exercise may attenuate EICF. Indeed, the elite underwent a longer duration and harder boot camp; all of these factors which are related with an increase in EICF (2). Nevertheless, the elite group demonstrated less post-acute prolonged endurance exercise-induced modifications. As age does not seem to play a significant role in EICF (2), the difference observed between the two groups studied can be related to the different training level of the soldiers. Indeed, the elite subjects were older and more used to these kind of selection programs, they were also more physically trained, as shown by larger left and right ventricular dimensions. This new finding might be in contradiction with two previous studies, which demonstrated that acute atrial and ventricular response to exercise were independent of training load (24) and cardiorespiratory fitness (i.e., peak$\overset{∙}{\;V}$O_2_ and training mileage) (33). An alternative hypothesis might be that the elite were already selected on their resistance to EICF, as they had already undergone several physical selection tests to join the elite.

### The Cascade of EICF

Another interesting finding is that deformation imaging seems more sensitive than conventional echocardiographic parameters for assessing EICF. Thus, the echocardiographic cascade of alterations indicative of EICF seems to begin with an alteration of speckle tracking derived parameters of diastolic function (i.e., atrial reservoir function) observed in both studied groups, then by an alteration of speckle tracking derived parameters of systolic function (i.e., RV and LV deformation, LV GCW, and lately mechanical dispersion), then by an alteration of the conventional markers of the diastolic function (i.e., E/A, E-wave deceleration time, e', and E/e') only observed in the non-elite group, and finally by an alteration of the conventional markers of systolic function (LVEF, RV FAC, TAPSE, mitral, and tricuspid waves) which was not observed in this study but reported in previous ones (4–6).

### Limits of the Study

We acknowledge several limits in this study. First of all, as already stated, the boot camp protocols were different. The boot camps were part of the usual military preparation, we were therefore not able to change them. But these programs were adapted to the abilities and training level of both populations studied.

For technical reason we were not able to weigh the elite subjects. The weight could be useful to estimate the LV preload but we recall that the systolic BP was not different before and after acute endurance exercise in this group and that the hydration was free for these high-experienced soldiers.

Lastly, the lack of follow-up represents a limit to describe evolutions of the observed alterations but pre-exercise echocardiograms were normal, and several previous studies have shown that the echocardiographic alterations that we reported were transient (1).

## Conclusions

Our study confirms that intense exercise lasting several days without interruption during boot camps induces cardiac fatigue, which is best detected with the 2D strain parameters of systolic and diastolic functions. Alteration occurred in both groups, even in the more experienced one. Nevertheless, there might by a relative protective effect of training level, as diastolic function and LV mechanical dispersion were only altered in the less trained group.

## Data Availability Statement

The raw data supporting the conclusions of this article will be made available by the authors, without undue reservation.

## Ethics Statement

The studies involving human participants were reviewed and approved by Rennes University Hospital and Regional Ethics Committee (Number 35RC13_8801). The patients/participants provided their written informed consent to participate in this study.

## Author Contributions

FS, FC, and DM: experiment conception and design. FS, GK, DM, and EG: experiments performed. MC and FS: data analyzed. MC, FS, FC, SL, TL, and ED wrote the paper. All authors read and approved the final manuscript.

## Conflict of Interest

The authors declare that the research was conducted in the absence of any commercial or financial relationships that could be construed as a potential conflict of interest.

## Publisher's Note

All claims expressed in this article are solely those of the authors and do not necessarily represent those of their affiliated organizations, or those of the publisher, the editors and the reviewers. Any product that may be evaluated in this article, or claim that may be made by its manufacturer, is not guaranteed or endorsed by the publisher.
